# Supplementary material for: Sulf2a controls Shh-dependent neural fate specification in the developing spinal cord
Source: Sci Rep. 2021 Jan 8;11:118. doi: 10.1038/s41598-020-80455-2 (PMC7794431; doi:10.1038/s41598-020-80455-2)
Supplement: Supplementary file 1 — Supplementary Figures. [file 41598_2020_80455_MOESM1_ESM.pdf]

TITLE PAGE

Sulf2a controls Shh-dependent neural fate specification in the developing spinal cord

Cathy Danesin*, Romain Darche-Gabinaud, Nathalie Escalas, Vanessa Bouguetoch, Philippe Cochard, Amir Al Oustah, David Ohayon, Bruno Glise and Cathy Soula.

Author affiliation

Centre de Biologie Intégrative (CBI), Centre de Biologie du Développement (CBD), Université de Toulouse, CNRS (UMR 5547), Toulouse, France.

Corresponding author information

* cathy.danesin@univ-tlse3.fr

Centre de Biologie Intégrative (CBI), Centre de Biologie du Développement (CBD), Université de Toulouse, CNRS (UMR 5547), Toulouse, France.

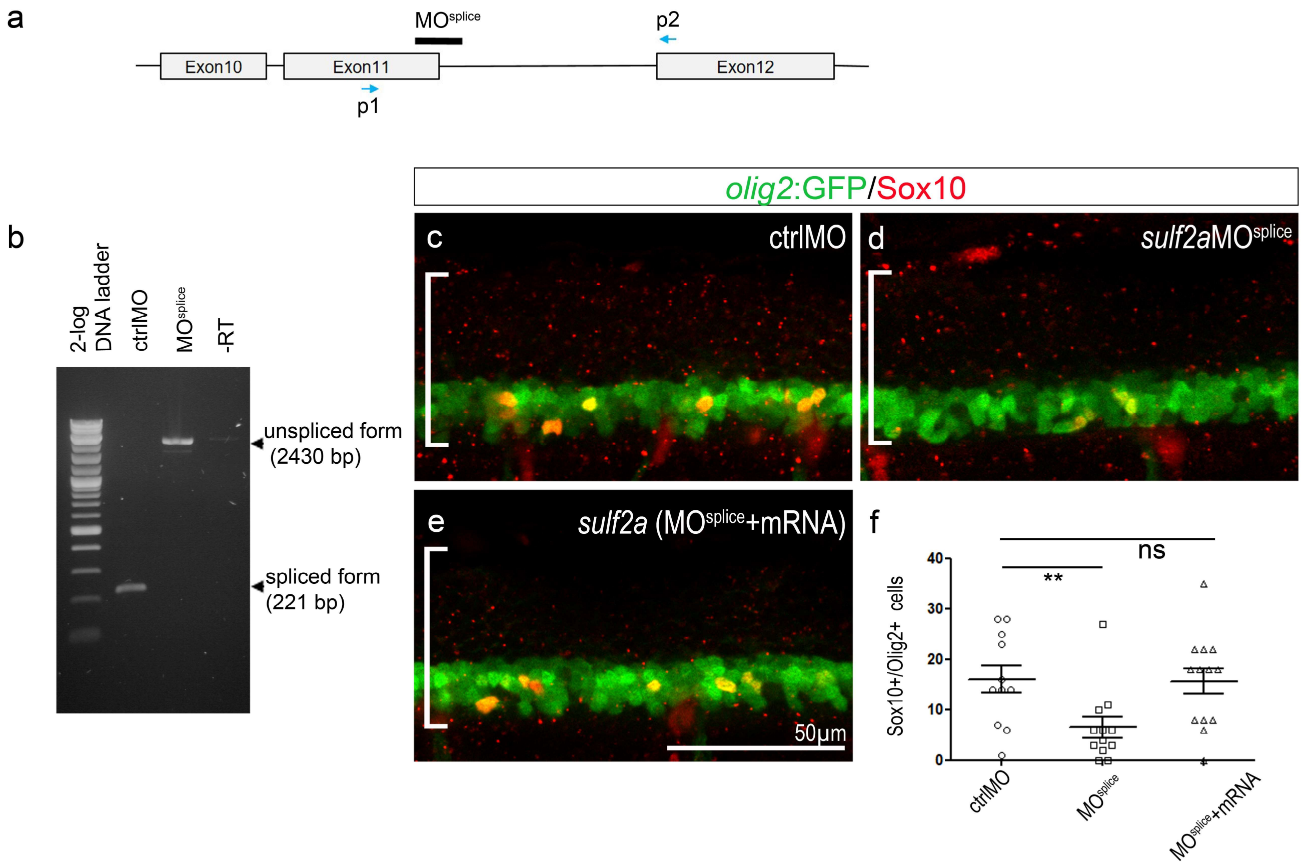

Supplementary Figure S1. Efficiency and specificity of *Sulf2a*MO^{splice} -mediated knockdown.

a: Schematic representation of *sulf2a*MO^{splice} target sequence overlapping *sulf2a* exon11.

b: Knockdown efficiency was controlled by RT-PCR at 24 hpf in embryos injected with ctrlMO or *sulf2a*MO^{splice} with primers p1 and p2 indicated on the scheme in a. A PCR product of 221bp evidences *sulf2a* splicing while 2430 bp sized PCR product testifies the presence of unspliced *sulf2a* pre-mRNA forms. PCR reactions were controlled on RNA extracts (-RT).

c-e: Side views of 48 hpf embryos. Detection (c-e) and quantification (f) of OPC by immunodetection of Sox10 (red) and GFP (green) in Tg(*olig2*:GFP) embryos injected with ctrlMO (c, n=11), *sulf2a*MO^{splice} (d, n=12) or *sulf2a*MO^{splice} +mRNA (e, n=13).

Datasets were compared using Mann-Whitney's test (two-tailed).

Data are presented as mean number of cells per embryo +/- s.d (** $p<0.01$, ns: not significant).

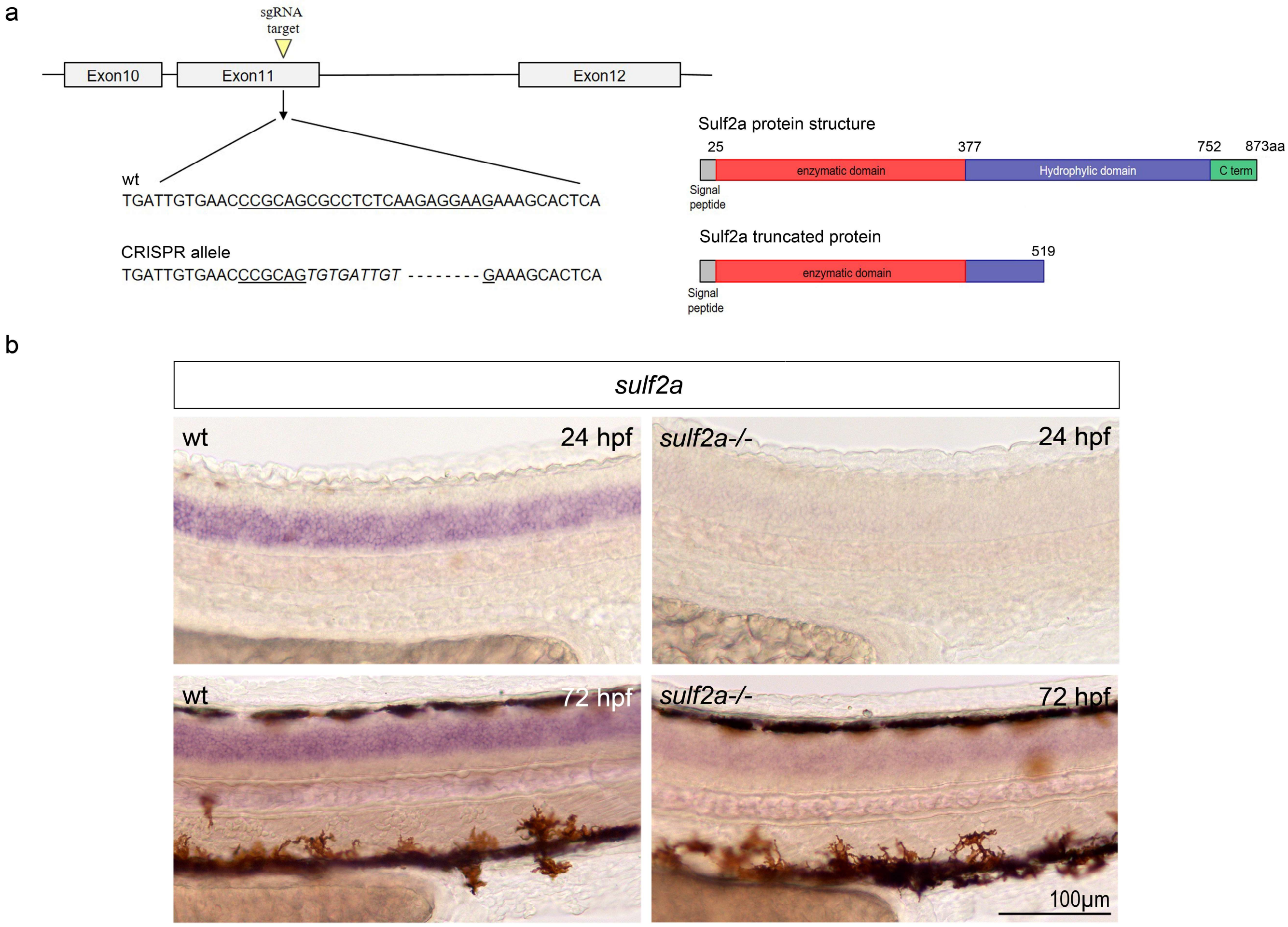

Supplementary Figure S2. Generation of *sulf2a* mutation by CRISPR/Cas9 technology.
a: Schematic representation of target sequence in *sulf2a* gene. The sequence targeted by CRISPR/Cas9-mutagenesis is indicated by a yellow arrowhead in exon 11.The wild type (wt) CRISPR target sequence is underlined and the mutant allele, consisting of a 16 nucleotide deletion/9 nucleotide insertion, is shown in italicand dashes.
This mutation is predicted to produce a 519 amino acid protein truncated within the hydrophilic domain. b: Lateral views of 24 hpf (top panels) and 72 hpf (bottom panels) embryos showing detection of *sulf2a* mRNA by whole-mount *in situ* hybridisation in wt (left panels) and *sulf2a-/-* (right panels) embryos. Note the decreased sulf2a expression in mutant embryos (n=10 at 24 hpf and n=6 at 72 hpf) compared to wt siblings (n= 7 at 24 hpf and n=8 at 72 hpf).

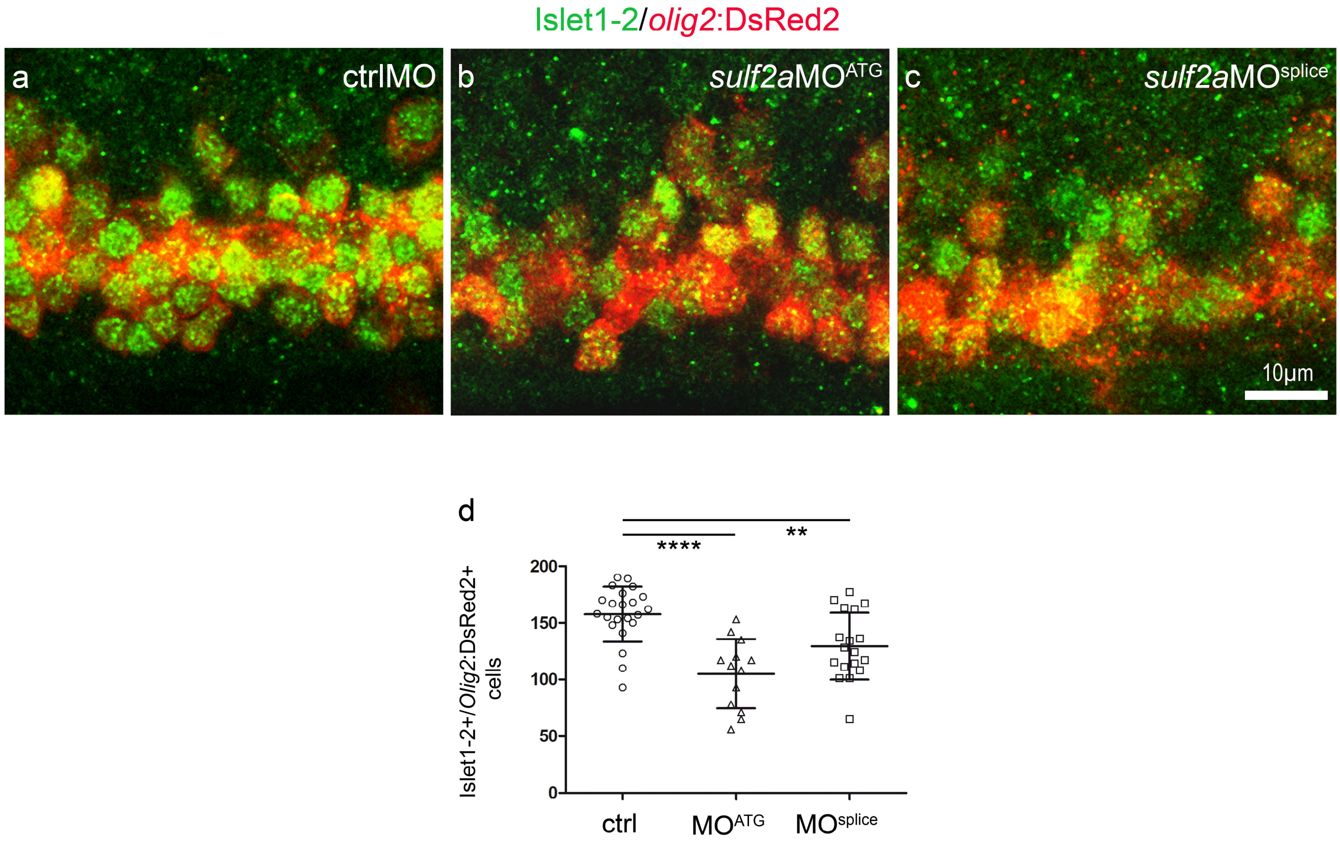

Supplementary Figure 3: *Sulf2a* depletion impairs motor neuron production
a-c: Detection (a-c) and quantification (d) of MNs by immunodetection of Islet1/2 (green) in Tg(*olig2*:DsRed) embryos (red) injected with ctrlMO (n=22), *sulf2a*MO^{ATG} (n=13) or *sulf2a*MO^{splice} (n=18) from two independent experiments. Datasets were compared with Mann Whitney's test (two-tailed). Data are presented as mean number of cells per embryo± s.d (**p< 0.01, **** p<0.0001).

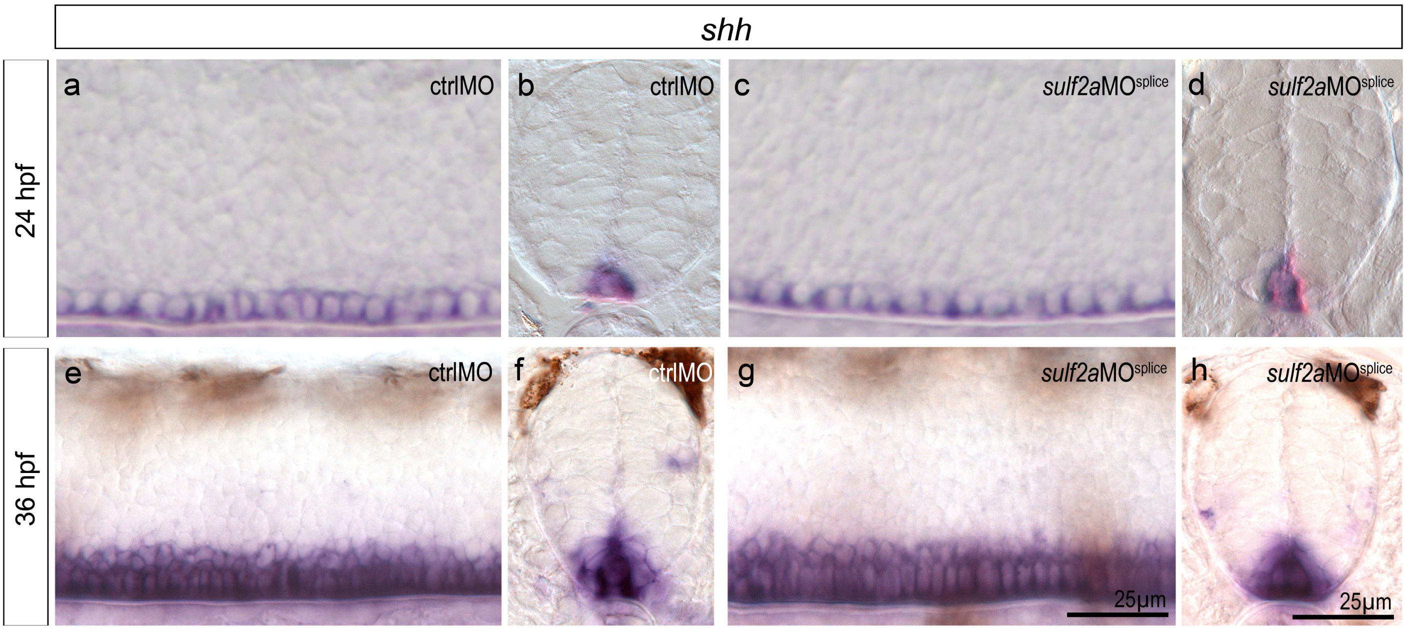

Supplementary Figure 4: *Sulf2a* does not regulate *shh* expression
Side views (a, c, e, g) and transverse sections (b, d, f, h) of 24 hpf (a-d) and 36 hpf (e-h) embryos. Detection of *shh* mRNA in embryos injected with ctrlMO (a, b, e, f) or *sulf2a*MO^{splice} (c, d, g, h).
